# Observations on Some Important Points in the Practice of Military Surgery, and in the Arrangement and Police of Hospitals: Illustrated by Dissections

**Published:** 1818-03

**Authors:** 


*Observations on some Important Points in the Practice of Military Surgery, and in the Arrangement and Police of Hospitals; Illustrated by Dissections.* By John Hennen, Deputy Inspector of Hospitals. pp. 520. *Edinburgh, London, Dublin.* 1818.

"Les circonstances," says a recent French writer on mi-

---

litary surgery, " qui contribuent le plus à la destruction des hommes, sont aussi celles qui font decouvrir et devellopent plus de moyens propres á leur conservation." Mr. Hennen's work is an excellent exemplification of this important and consoling truth. If war has destroyed its *millions*, it will at least save its *thousands*. But this is a contracted view of the influence which warfare has had on the improvements of medical and surgical science. The evils are temporary, the good is permanent. When the victories of a Wellington are forgotten, the triumph of military surgery will descend from age to age, " with healing on its wings;" the brightest gem in the crown of science,—the surest resource in the hour of peril and suffering.

Mr. Hennen's work is the result of wide experience, accurate observation, and unwearied zeal. It is, therefore, one which will be found useful to all, and *essentially necessary* to a large class of medical and chirurgical practitioners. To the army and navy surgeon it needs not be recommended, for they can too well appreciate its importance, to hesitate a moment in possessing themselves of the book.

" In the foregoing pages, (says our author), I have, as far as I was capable, divested myself of every prejudice, and been the faithful narrator of what I have seen. I have neither indulged in the visions of theory nor the intricacies of criticism, nor have I strained or distorted facts to serve a particular purpose." 491.

In the Introduction, Mr. Hennen draws an animated sketch of the history of military surgery, which evinces much judicious research, and proves, that while his principal business has been among *men* he has not neglected *books*. In arranging his materials,

" He has carefully availed himself of the written and oral remarks of the best army surgeons, both domestic and foreign, to whose works or conversation he has had access, or who have had more experience than himself."

This is wise conduct. The man who aims at exclusive originality in these days, may excite the wonder of a *few*, but will not prove useful to the *many*.

The following passage is very appropriate to the present rage for extravagant eccentricity in new remedies and wild theories.

" The military surgeon must, to the habits of subordination, add professional enthusiasm:—not that ungoverned impulse which catches at the ephemeral proposals of empty theorists or self-dubbed reformers, but that regulated and chastened zeal, which has real and useful acquirements for its object, and which, while it strengthens and increases his own powers and resources, gives him confidence in his principles, and an honest independence in their application." 18.

Mr. H. next lays down a code of clear and excellent directions for the conduct of the young military surgeon on taking the field, and opens the immediate subject of the work by an able dissertation on "the general Nature and first Treatment of Wounds, Course of Balls, &c." 30. As every page is pregnant with interesting matter, and practical observations, a regular analysis of a work of this kind is impossible, without transcribing the whole book. We can do little more than offer a few samples from the different sections; but this we assert, that we know not which to choose, since all is excellent, and nothing irrelevant. Speaking of the course which balls occasionally take, Mr. H. among numerous interesting instances, relates the following.

"In a case which occurred to a friend of mine in the Mediterranean, the ball, which struck about the pomum Adami, was found lying in the very orifice of its entrance, having gone completely round the neck, and being prevented from passing out by the elasticity and toughness of the skin, which confined it to this circular course." p. 33.

Many ingenious observations are made by Mr. H. on the propensity of balls to course round a concave surface, as the interior of the peritoneum and pleura, often without injuring the contained viscera: this propensity is also seen in cases where the ball strikes the front of a hat, and running round, carries off the hinder tassel.

"If the ball has fairly passed through the parietes of the thorax or abdomen, we dress both orifices, as in the first case, and take away from sixteen to twenty-four ounces of blood from the arm, if no hæmorrhage has followed. We should be equally attentive to the abstraction of blood in cases where a round shot, or piece of shell has grazed the head, neck, thorax, abdomen, or any of the joints; or where they have been contused by the splinters of a shell, or flying stones, or clods of earth. In all these cases we shall also derive much future benefit from unloading the bowels by a calomel pill, with some antimonial powder; a medicine both from its purgative power and its portability, which should always be ready in the field panniers." p. 38.

Clear and explicit directions follow, as to the management of gunshot wounds on the field, which ought to be studied, and even committed to memory by the army and navy surgeon. In respect to the question of amputating immediately after the infliction of a wound, or consecutively, Mr. H. observes, that the practical reply is: *with as little delay as possible*." Yet Mr. H. here, and always indeed, combines caution with decision: the surgeon, he remarks, "will betray a miserable want of science, if, in the crowd of sufferers, he indiscriminately amputates the weak, the terrified, the sinking, and the determined." 49. If he find a patient with feeble concentrated pulse, faintness, loss of reason, convulsions, hiccup, vomiting, coldness, and other marks of violent derangement in the balance of the circulation, and excitement, he will wisely endeavour to restore, as far as possible, the equilibrium of both, by a cautious administration of wine, warmth, volatile alkali, soothing encouragement, and *time* before he proceed to the operation.

The duties and arrangements of the receiving hospital are next pourtrayed with the hand of a master, and with that facility of description which evinces the perfect acquaintance with every minutia of the subject.

Mr. Hennen justly remarks, that whatever opinion may exist to the contrary, soldiers bear phlebotomy better than any class of people in private life: the whole tenor of their diet, exercise, and discipline tends to carry their system to the highest possible pitch of vigour, when effective in the field; and there they will bear an extent of evacuation, which they could not possibly have stood up against in their native fields, with all the appearance of ruddy, rustic health.

Mr. H. conceives that few, if any, of the *veterans* are without either confirmed hepatic affections, or a strong tendency that way. This may be easily accounted for by the irresistible propensity which soldiers and sailors have ever evinced towards intemperance, when out of the inspection of their officers, together with their exposure to all the inclemencies of the atmosphere, &c. It is on this account that double attention is necessary to the functions of the epigastric viscera, during the irritation of wounds; a degeneration of their discharges, and a deranged state of the chylopoetic organs are inseparably connected, the former however, very frequently dependent on the latter. The functions of the skin too, are decisively influenced by, and influence in their turn, the state of the primæ viæ, and consecutively of the wounds.

On the subject of extracting foreign bodies, every thing is said that can be well said, and various interesting cases in illustration introduced. The following is equally curious and interesting.

"A Hanoverian soldierreceived a severe wound from a grape shot, on the 18th June, 1815, at Waterloo, which struck him on the external part of the thigh, producing very extensive laceration. On the second day he was brought into the hospital, and the usual dressings applied. On the fifth day, a long narrow passage was discovered by the probe, seeming to run nearly the whole length of the vastus externus muscle. On cutting into this, three pieces of coin (which, from the very curious mode in which they were compacted together, I thought worthy of presenting to the Director General of Hospitals), were extracted from the parts. This poor fellow, a raw recruit, had no money whatever about him, nor even a pocket to contain it in, and fervently protested against his right to this forced loan. He accounted for it, by supposing it carried from the pocket of a comrade, who stood before him in the ranks, and who was killed by the same shot.

"The coins, consisting of two five-franc pieces and a Dutch stiver, were obviously first struck by the shot, and carried along by it; for nearly one half of their flat surfaces, the silver pieces adhered closely together; on the other, where the ball had struck their edges, the metal was flattened out, and somewhat hollowed. In this hollow lay the copper coin; in some degree adapted to the shape of the depression on the larger pieces.

"I cannot omit noticing here a trait strongly illustrative of the mobility of mind which characterizes soldiers, and their proneness to superstition and belief in omens, which a surgeon, acquainted with their character, can often turn to their benefit. The part of those two coins which had been flattened out, happened to be that on which Napoleon's head was impressed. From one it was nearly effaced; and on observing this circumstance to the patient and his comrades, an universal burst of joy echoed through the ward. The young Hanoverian exulted in the share he conceived he had personally had of contributing to the downfall of the French Emperor. His health rapidly improved, and I have no doubt this simple circumstance had a good effect upon every man who witnessed it." p. 89.

The section on Contusions, and other serious injuries from shot and shell, is very interesting, and contains a selection of cases, well calculated to illustrate all the admirable practical precepts laid down by our author. The succeeding section on "Injuries of the Bones," is still more important. Some information, Mr. H. observes, may often be gained from the appearance of the ball itself. Where it has brushed obliquely past a bone, and injured its external plate, its surface is often jagged, and presents the appearance of a file, clogged with raspings of ivory: sometimes the ball is firmly fixed in the bone, and can only be removed by sawing the bone with the crown of a trephine: the accident is always serious; but it is possible under circumstances of peculiar good fortune in a temperate subject of sound constitution, to save the limb by the operation, as in the following case.

"Joze de Santos, a quarter-master in the 19th Caçadores, passing along the bridge of Burgos, on the 27th September, 1812, was struck by a musket ball on the outside of the knee, which brought him to the ground. "I found him," says Staff Surgeon Hughes, "at the Hospital del Rey, about three hours and a half afterwards, in great pain; the parts surrounding the joint swelling rapidly, and a Portuguese surgeon endeavouring to persuade him to suffer amputation. The ball was lodged and could not be found, although the wound had been a little dilated, to facilitate examination. Measures to subdue inflammation were immediately adopted. On the morning of the 28th, he had a violent shivering fit, and another at midnight; copious suppuration was found to have taken place on the 29th, with an abatement of pain, and the ball was easily felt, but immoveable, and seemingly stuck in the bone. Poultices and fomentations were now applied; and on the 1st of October, I found he had had another shivering fit the preceding night, and that a piece of cloth had come away with the discharge, which was much increased. This evening he was attacked with diarrhœa, and vomited some bilious matter; the suppuration now became profuse, the diarrhœa grew progressively worse, and his rigors continued to return with an exhausting purulent discharge, until the 7th, when amputation was again proposed, as the only means of preventing a rapidly, fatal termination; but he persevered in his resolution, to prefer death to this operation. As the only alternative, I extensively dilated the wound down to the bone, when the ball was found fixed in the centre of the external condyle of the femur, nearly a quarter of an inch below the bony surface. The crown of a trephine was now applied, and a flattened ball was extracted, with several portions of cloth: light dressing was applied, and next morning it was found he had escaped his shivering fit; his diarrhœa was abated, and he had enjoyed sleep; which, since the first day of his wound, had been nearly a stranger to him. The discharge continued gradually to decrease, and his health to improve; and on the 22d, (when circumstances caused the removal of the wounded,) the parts were healing readily, and anchyloses taking place. He bore his journey in a waggon well; and, when discharged from the service three months after, was in good health, and had a tolerably straight knee." p. 115.

Respecting the *position* in simple or compound fractures of the thigh, Mr. Hennen gives the preference, upon the whole, to the French plan, namely, extension of the limb, and position on the back, which he says is by far the most tolerable to the patient, and admits of much easier access and dressing. "Its ultimate success is equal, if not superior to either the bent position of Pott, the patient on his side, or the semiflexion of the knee, the patient on his back, and the limb in a fracture box, as recommended by Mr. Charles Bell and others." 119. But to this section we cannot do any justice, as it is very long, and very valuable in every part.

The section on "Injuries of the Joints" is also extremely interesting. In Mr. H's own practice, he has only met with two cases, where the limb was saved after serious injury of the knee joint; and in one of them only was the perfect use of the member restored. The injuries of the ankle and elbow joints are equally unfavourable; shoulder joint cases are more favourable than either of the above. At page 162, *et seq.* is related a most interesting case of Lieut. Col. R. of the Dragoons, who received a musket shot in the right knee joint, in the battle of Waterloo: the extent to which general and local bleeding was carried, together with rigid abstinence, would surprise some of our private practitioners; but the means were ultimately successful: the bullet was never extracted.

*Injuries of the Blood-vessels.* This is a division of the work which deserves the serious study of every chirurgical practitioner. Mr. H. truly remarks, that how the blood-vessels escape so often as they do, is truly wonderful. Balls buff along them, pass between them, and traverse their courses in all directions, yet frequently leave them unhurt. Their great elasticity must be the grand cause of this; and we cannot coincide with our author, that "something also may depend upon their being in a state of dilatation or otherwise when the missile weapon passes across them." 179. The vessels are always full while life remains, and it is now pretty generally admitted, that no dilatation or contraction corresponds with the systole and diastole of the cardiac ventricles.

Mr. H. truly observes, that it is one thing to cut down upon a sound artery in the living or dead body; but another to cut down upon, find, and tie a wounded one. The difficulty, in the latter case, is owing, of course, to the engorgement produced by the extravasated blood and other fluids among the interstitial spaces, destroying all relation of position as found in sound parts. It appears from Mr. Hennen's candid account of the business, that the plan of cutting off both ends of the ligature, leaving the knot to find its way out, or dissolve and be absorbed, first originated with a practitioner, Dr. Maxwell, of Dumfries, in 1798. That in the year 1813, it was suggested to Mr. Hennen by Assistant Staff Surgeon Hume, and put in practice in Spain with success. Mr. H. afterwards published an account of the affair in the third vol. of the London Medical Repository. The battle of Waterloo furnished our author with additional evidence of the utility of this plan, of which he speaks in high terms of commendation. The following operation lately occurred in Edinburgh-Castle.

"William Bisset was admitted into hospital on the 1st of December 1816, with an extensive, irritable, and sloughing bubo in the groin. On the 26th of the month, the external pudic artery, which was involved in the ulceration, burst, and discharged about a pint of blood, which was restrained by pressure; a second hæmorrhage took place next day, and the ulceration spread still farther. On the 31st, the blood sprung from the artery in a full jet, when the actual cautery and pressure restrained it. On the 12th of January, the hæmorrhage again returned, and was controlled by pressure. It continued to recur so often from this period, that the life of the patient was in imminent danger, until, on the 22d a dreadful discharge of blood threatened at once to terminate his existence. Constant pressure was now applied, and, next day, on consultation, the parts were accurately examined, when, on removing the clots, it was found that the femoral artery itself had given way. No other resource now remained but tying the external iliac, which was accordingly done as follows: An incision was made between three and four inches long, through the integuments in the direction of the artery, beginning at Poupart's ligament, and carrying it upwards. The lower edge of the internal oblique and transverse muscles being divided, the artery was exposed, which being, with some difficulty, separated from the surrounding parts and from the vein, a blunt curved needle was passed under it, a single ligature applied, and the edges of the wound brought together. On the 24th, the limb felt cold and was insensible, except when firmly pressed upon. On the 25th, the skin about the knee became discoloured, and a copious sanious discharge took place from the wound. On the 26th the discolouration extended, a vesicle formed about the centre of the thigh, and a considerable quantity of coagula and sanies was removed from the groin. On the 31st, the leg was extensively mortified. On the 1st of February, tension and pain of the abdomen came on, which was relieved by a dose of castor oil. On the 2d, separation in various parts of the thigh began to establish itself. On the 9th, amputation was performed at a point close up to the trochanter, every thing went on well afterward, and the man perfectly recovered." P. 199.

*Injuries of the Nerves.* Mr. H. has seen several cases where the nerves had been injured in their passage down the humerus, and the extremities alone of the fingers suffered, all the intermediate parts possessing their full powers; and others where pressure on the crural nerve close to its exit from the body has affected one or two of the toes only. With the following curious and interesting case we shall close our analytical sketch of the first half of the volume before us.

Case. "A general officer, of distinguished gallantry, was struck by a round shot during a very desperately fought action, which, buffing along his breast in an oblique direction, destroyed the arm, and left only the head of the bone and a very small portion of the shaft remaining. He was carried to an adjoining hovel, where the common amputation was performed under very unfavourable circumstances; the night was coming on, the supply of candles was scanty, and the enemy's shot were flying in all directions. The General was placed under my care the day after the operation. The variety of cross accidents from fever and extensive sloughing, it is not within my purpose at present to enlarge upon, but the first attempt at clearing the ligatures, and making gentle pressure on them, was attended with pain so excruciating, as to leave no doubt that each included a nerve, or was in a certain degree connected with some large nervous filaments. This agonizing sensation was not felt, except the ligatures were pulled at, and then not in the stump itself, but referred to the finger, thumb, wrist, elbow, or even to the external skin of the lost arm, as one or other ligature might be handled. I have sometimes been led to think, that the General uniformly felt the same sensations when the same ligature was touched, as I generally made my attempts to extricate them in a regulated succession, and his complaints were often of the same succession of parts. More attentive observation, however, convinced me that this was not the case; for if any one was pulled with more steadiness than another, he complained of all the parts suffering pain simultaneously. One small ligature if pulled in an oblique direction *inwards* towards the axilla, always gave him imaginary pain about the elbow or in the skin; but if the same was pulled strongly and directly *downwards*, the fingers were complained of. He has frequently after the smarting of dressing was over, with great accuracy pointed out the course of the internal cutaneous nerve, as the site of his ideal pain; often he has described that of the external; and, on one occasion, I, with utter astonishment, had the general neurology of the arm and fingers traced by him. But unless the ligatures were pulled at, he had no other uneasy sensations than those which usually occur in persons whose limbs have been amputated. Once only did I ever know him refer his pain to the seat of the sensorium itself. On that occasion, from using an artery forceps to the ligatures, on which the slide moved rather stiffly, I exerted a greater force than I intended. He convulsively put his hand to his head, expressed a sense of exquisite pain in his brain, involuntary tears dropped from his eyes, a paralytic contraction momentarily affected his mouth, an universal paleness spread over the uncovered parts of the body; and though unusually tolerant of pain, and of a most remarkable equanimity of temper, he uttered a piercing cry, and exclaimed, " that the agony in his head and neck was insufferable." The state of collapse was so great, that I was obliged to send an aid-de-camp instantly for volatile alkali, and a glass of Madeira, by which he was soon relieved; but the painful sensation, and the prostration of strength, continued through the day. A British admiral was present on this and various other occasions, and observed to me, after I had confessed my inability to explain, even to my own satisfaction, the cause of all these sensations, " that he never saw the General dressed without applying mentally to the wonderful sympathy manifested on those occasions, the expression of Pope 'it lives along the line.'—I believe we must be content with the fact, without seeking for the explanation." P. 206.

We have been able to do no kind of justice to this interesting volume, in our present rapid analytical view of a part of its contents; and we confidently expect that, ere our labours are closed in the ensuing Number, most of our readers will have perused the original. We have no hesitation in affirming, that this is one of the most useful and valuable practical works in Surgery that has appeared in the English, or in any other language.

---

*Surgical Observations, Part IV.* By Charles Bell.

